# Genome-Wide Characterization of the WOX Gene Family in *Polygonatum cyrtonema* and Its Applications for Regeneration

**DOI:** 10.3390/plants15111745

**Published:** 2026-06-04

**Authors:** Yan Shi, Binjie Huang, Cong Li, Jinping Si, Donghong Chen

**Affiliations:** State Key Laboratory for Development and Utilization of Forest Food Resources, Zhejiang A&F University, Hangzhou 311300, China; sy2023@zafu.edu.cn (Y.S.); 13968855750@163.com (B.H.); lenilc@163.com (C.L.)

**Keywords:** gene expression, subcellular localization, shoot induction, rooting, transcription factor

## Abstract

*Polygonatum cyrtonema* is a medicinally and edible perennial herb, yet functional studies in this species are constrained by limited knowledge of regeneration-associated developmental regulators. Here, we performed a genome-wide characterization of the WUSCHEL-related homeobox (WOX) gene family in *P. cyrtonema*. Eleven *PcWOX* genes were identified and classified into the ancient, intermediate, and modern/WUS clades. Comparative phylogenetic, syntenic, structural, and chromosomal analyses indicated that the *PcWOX* family retains a conserved evolutionary framework but also exhibits clear lineage-specific diversification. Tissue- and stage-specific expression profiling, promoter cis-element analysis, and subcellular localization further supported functional differentiation among *PcWOX* members, particularly between *PcWOX5* and *PcWUS*. Weighted gene co-expression network analysis identified 33 co-expression modules, including six key modules strongly associated with *PcWOX* abundance patterns, and prioritized 49 candidate transcription factors (TFs) to construct *PcWOX*-centered regulatory networks. These TFs showed marked tissue- and stage-dependent heterogeneity. Heterologous assays in *Nicotiana benthamiana* showed that both *PcWUS* and *PcWOX5* enhanced regeneration competence but produced distinct developmental outputs. These findings support *PcWUS* and *PcWOX5* as promising candidate morphogenic regulators and provide a useful framework for future mechanistic studies, homologous validation, and regeneration improvement in *P. cyrtonema*.

## 1. Introduction

**Figure 1:**
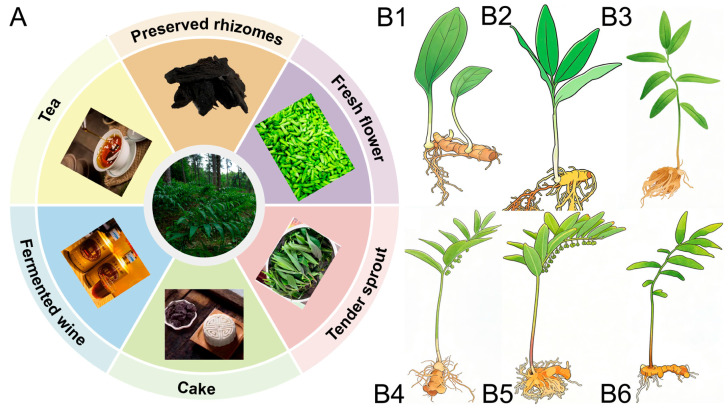
Food and medicinal value and developmental stages of *P. cyrtonema*. (**A**) Food and medicinal uses of *P. cyrtonema*. (**B**) Representative images of *P. cyrtonema* at six developmental stages (**B1**–**B6**, corresponding to stages 1–6; S1–S6).

*Polygonatum cyrtonema* Hua (Huang Jing) is a perennial herb that has been widely used in Asia for centuries as both a medicinal plant and a functional food because of its health-promoting properties [1]. Its rhizomes are rich in bioactive compounds, including polysaccharides, steroidal saponins, and flavonoids, which contribute to diverse pharmacological activities, including anti-inflammatory, hypoglycemic, lipid-regulating and other physiological effects [2]. Traditional processing through nine cycles of steaming and drying transforms fresh rhizomes into sweetened preserved products, allowing dual applications: direct consumption and functional incorporation into foods (e.g., baked goods) to enhance sensory and nutritional profiles (Figure 1A) [3]. With the increasing market demand for *P. cyrtonema*, efficient propagation and genetic improvement of this species have become increasingly important. However, despite its economic and medicinal value, molecular studies in *P. cyrtonema* still lag far behind those in model and major crop species [4]. Although multi-omics analyses have begun to reveal pathways associated with metabolite accumulation in the *Polygonatum* genus, progress from correlation to causation remains constrained by the limited availability of robust regeneration- and transformation-related platforms. Recent studies in the *Polygonatum* genus, especially in *P. cyrtonema*, have advanced regeneration-related research in several directions, including tissue culture propagation, micro rhizome induction, preliminary transformation-related optimization, and functional assays based on transient expression, VIGS, and hairy-root systems [5,6,7]. In addition, an indirect somatic embryogenesis and plant regeneration system using immature embryos as explants has recently been established in *P. cyrtonema*, representing a major advance in in vitro regeneration [7]. However, existing platforms are still mainly designed for propagation, transient assays, VIGS, or hairy-root induction, and are often highly dependent on explant type, genotype, and culture conditions [6,7]. As a result, a stable and broadly applicable regeneration/transformation framework for gene functional analysis in *P. cyrtonema* is still lacking. In this context, identifying developmental regulators that can enhance regeneration competence is of particular importance for future transformation-system improvement and molecular breeding in this species.

The WUSCHEL-related homeobox (WOX) transcription factor family encodes plant-specific homeobox transcription factors that regulate stem cell maintenance, organogenesis and embryogenesis [8,9]. WOX proteins are generally grouped into ancient, intermediate and modern (WUS) clades; modern-clade members such as WUS and WOX5 serve as master regulators of shoot and root meristems, respectively [10,11,12]. Overexpression of WOX genes has been used to promote regeneration and overcome genotype dependence in several species [13]. Importantly, overexpression of WOX factors can dramatically improve in vitro regeneration and transformation efficiency in crop species. For instance, ectopic expression of a wheat WOX5 ortholog (*TaWOX5*) greatly enhanced *Agrobacterium*-mediated transformation: transformation frequency increased from ~6% to over 50% in a normally recalcitrant wheat variety [14]. In apples and kiwifruits, overexpression of *MdWOX5* can increase the regeneration efficiency from 3.3% to 20.6% [15]. These findings illustrate that manipulating WOX genes can overcome genotype-dependent barriers and markedly boost regeneration and transgenic plant production. Therefore, characterization of *PcWOX* genes and leveraging their function could facilitate transformation and regeneration in *P. cyrtonema*.

*Agrobacterium*-mediated transformation is a fundamental tool in plant biotechnology, enabling stable integration of foreign T-DNA into plant genomes [16]. Although the molecular mechanism of T-DNA transfer is well established, transformation efficiency is strongly influenced by experimental parameters during infection and selection. Among these, the concentration of selection agents [17], *Agrobacterium* inoculum density [18], and infection and co-cultivation duration [19] are consistently identified as critical determinants of successful transformation. Inappropriate selection pressure may either fail to suppress bacterial overgrowth or severely inhibit plant cell viability, thereby reducing callus, while suboptimal bacterial density or infection time can compromise T-DNA delivery or cause phytotoxic effects due to bacterial overproliferation. These factors often interact in a species- and genotype-dependent manner, necessitating empirical optimization for each transformation system.

In this study, we performed a genome-wide characterization of the WOX gene family in *P. cyrtonema* and examined their phylogeny, structural features, promoter architecture, expression patterns, and subcellular localization. We further used weighted gene co-expression network analysis (WGCNA) to prioritize key modules and candidate transcription factors associated with *PcWOX*-centered regulatory networks, and assessed the regeneration-promoting potential of *PcWUS* and *PcWOX5* in a heterologous *Nicotiana benthamiana* leaf-disk system. Together, this work identifies candidate morphogenic regulators and provides a framework for future mechanistic studies, homologous functional validation, and the optimization of regeneration and genetic transformation strategies in *P. cyrtonema*.

## 2. Results

### 2.1. Identification and Phylogenetic Classification of the PcWOX Genes

**Figure 2:**
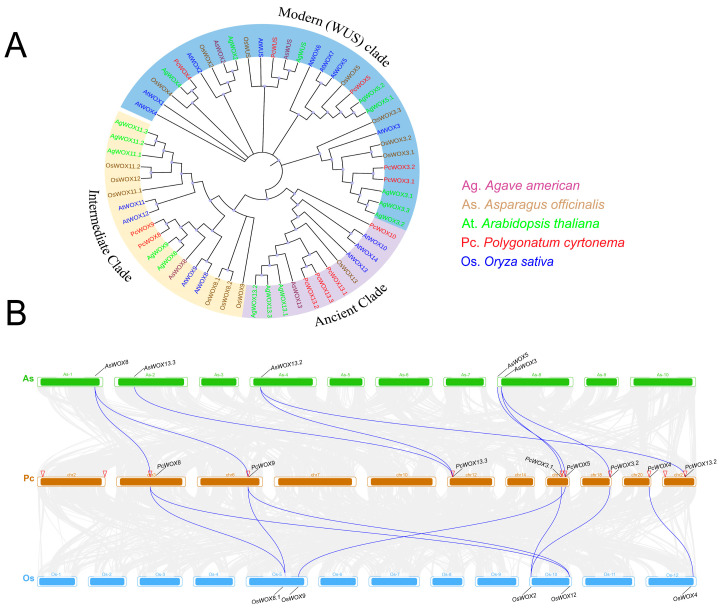
Phylogenetic classification and syntenic relationships of the *PcWOX* gene family. (**A**) Neighbor-joining phylogenetic tree constructed using WOX protein sequences from *P. cyrtonema*, *A. americana*, *A. officinalis*, *A. thaliana*, and *O. sativa*. Bootstrap support values were calculated from 1000 replicates and are shown at the nodes. (**B**) Synteny analysis of WOX genes among *P. cyrtonema*, *A. officinalis* and *O. sativa*.

A total of 11 *WOX* genes were identified in the *P. cyrtonema* genome. Based on phylogenetic analysis using WOX proteins from *P. cyrtonema*, *Arabidopsis thaliana*, *Agave americana*, *Asparagus officinalis*, and *Oryza sativa*, these *PcWOX* members were classified into three well-defined clades consistent with model species: the modern (WUS) clade (*PcWUS*, *PcWOX3.1/3.2*, *PcWOX4*, and *PcWOX5*), the intermediate clade (*PcWOX8* and *PcWOX9*), and the ancient clade (*PcWOX10* and *PcWOX13.1/13.2/13.3*) (Figure 2A). All WOX gene IDs used in the phylogenetic analysis are listed in Supplementary Table S2. Notably, members of the modern (WUS) clade, particularly *PcWUS* and *PcWOX5*, clustered closely with their respective orthologs in *A. officinalis* and *O. sativa*, suggesting conserved roles in meristem maintenance and organogenesis. In addition, the PcWOX proteins showed substantial variation in amino acid length, molecular weight, and isoelectric point, while most were predicted to localize to the nucleus, except PcWOX8, which was predicted to localize to the mitochondrion, thereby providing basic information on the physicochemical properties and subcellular localization of the PcWOX family (Supplementary Table S3).

To elucidate the expansion mechanisms of the WOX family, we performed a comparative syntenic analysis between *P. cyrtonema* and two other monocots, *A. officinalis* and *O. sativa* (Figure 2B). The results revealed varying degrees of gene expansion and conservation across these lineages. Specifically, the key regulatory members *PcWOX5* (Pc-chr16) and *PcWUS* exhibited remarkable evolutionary stability, with *PcWOX5* maintaining a strict 1:1:1 orthologous relationship with *AsWOX5* (As-chr8) and *OsWOX9* (Os-chr5). In contrast, other *PcWOX* members showed lineage-specific expansion through segmental or whole-genome duplication (WGD) events. For instance, in the intermediate clade, the *AsWOX8* (As-chr1) and *OsWOX8.1/12* (Os-chr5/10) loci corresponded to two *PcWOX* homologs, *PcWOX8* (Pc-chr3) and *PcWOX9* (Pc-chr6). Similarly, within the WUS clade, *PcWOX3.1* (Pc-chr16) and *PcWOX3.2* (Pc-chr18) were syntenic to single loci in both *A. officinalis* and *O. sativa*. These findings indicate that while core members like *PcWOX5* and *PcWUS* remain stable to preserve essential developmental functions, other subfamilies have diversified to potentially facilitate environmental adaptation in *P. cyrtonema*.

### 2.2. Gene Structure, Conserved Motif and Chromosomal Distribution

**Figure 3:**
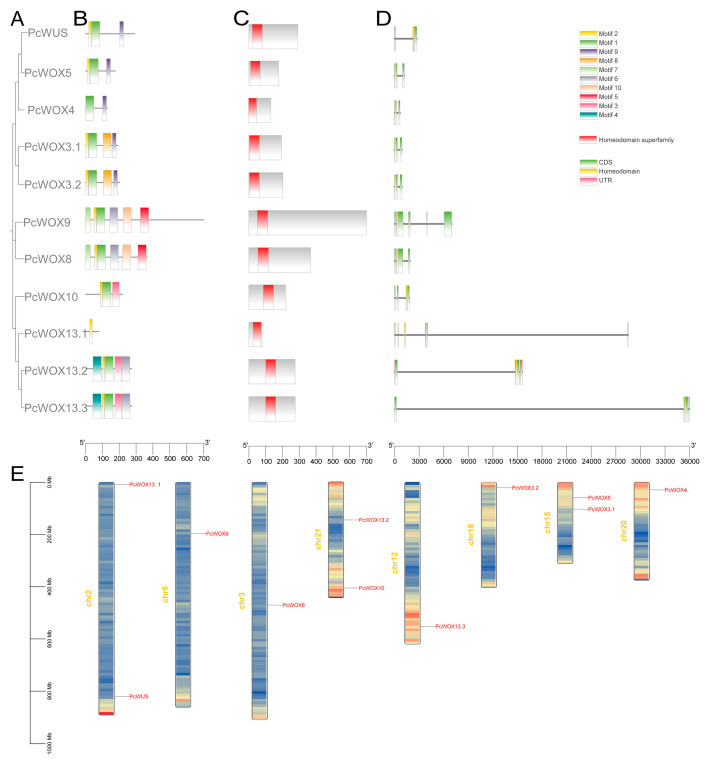
Gene structure, conserved motifs, conserved domains, and chromosomal distribution of *PcWOX* genes. (**A**) The phylogenetic tree of *PcWOX*. (**B**) The distribution of conserved motifs; (**C**) The conserved protein domains. (**D**) Gene structure. (**E**) Chromosomal mapping of *PcWOX* genes.

We identified eleven WOX family members in the *P. cyrtonema* genome. Phylogenetic placement showed that *PcWOX5* and *PcWUS* were closely related within the same subclade (Figure 3A). MEME analysis detected up to ten conserved motifs across the *PcWOX* proteins, and motif arrangement was largely conserved within subfamilies. For example, both *PcWOX5* and *PcWUS* contained three motifs, whereas *PcWOX8* and *PcWOX9* each contained six motifs (Figure 3B). Domain annotation confirmed that all intact *PcWOX* proteins carry the characteristic homeobox domain (Figure 3C). Exon–intron structures vary among family members but the homeobox-coding region is preserved in all genes with complete coding sequences; only *PcWOX13.2* retained annotated UTRs in our assembly (Figure 3D). Mapping *PcWOX* loci to the *P. cyrtonema* pseudochromosomes showed an uneven distribution: the eleven genes are located on eight chromosomes, with chromosomes 2, 15 and 21 each harboring two *PcWOX* genes, and chromosomes 3, 6, 12, 18 and 20 each containing one (Figure 3E). This dispersed pattern suggests a history of independent duplication and rearrangement events and provides a baseline for interpreting *PcWOX* evolution and potential positional effects on regulation.

### 2.3. The Expression Profiles, Promoters Cis-Element Composition, and Subcellular Localization of PcWOX Genes

**Figure 4:**
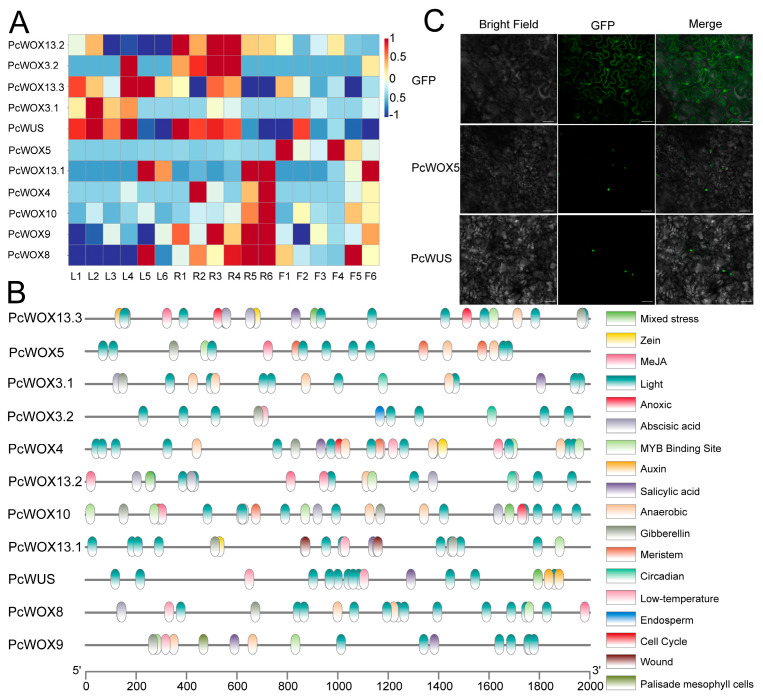
Tissue- and stage-specific expression patterns, promoter cis-regulatory elements, and subcellular localization of *PcWOX* genes. (**A**) Heatmap showing the expression patterns of *PcWOX* genes across different tissues and developmental stages. (**B**) Distribution of cis-element regulatory elements in the promoter regions of *PcWOX* genes in *P. cyrtonema*. (**C**) Subcellular localization of *PCWOX5*-GFP and *PCWUS*-GFP in *N. benthamiana* epidermal cells. GFP, green fluorescence channel; merge, overlay of GFP and bright-field images. Scale bar = 100 µm.

Heatmap analysis revealed distinct tissue- and stage-specific expression patterns of PcWOX genes in leaf, rhizome and fibrous root (Figure 4A). In leaf and rhizome, *PcWUS*, *PcWOX3.1*, *PcWOX13.3*, *PcWOX13.2* and *PcWOX3.2* increased progressively from Stage 1 (seedling) to Stage 4 (one month after flowering) and declined thereafter (Stages 5–6), whereas their transcript levels in fibrous root remained relatively stable across stages. *PcWOX5* was expressed predominantly in the fibrous root: its abundance fell sharply from Stage 1 to Stage 3 (flowering), rose markedly at Stage 4, and then decreased in later stages. The remaining five *PcWOX* members exhibited a gradual up-regulation from Stage 1 through Stage 6, reaching maximal expression in the rhizome at mid-fruiting (Stage 6; 90 days post-flowering). Across Stage 1–6, expression levels ranked rhizome > fibrous root > leaf for these genes (Figure 4A). Differential expression analysis (|log2FC| ≥ 1, Padj < 0.05) identified stage- and tissue-specific DEGs within the *PcWOX* family. Read counts for *PcWOX* genes were normalized using the Trimmed Mean of M-values (TMM) method implemented in edgeR to obtain expression values for heatmap visualization (Supplementary Table S4).

Promoter scanning (2 kb upstream) identified 18 types of cis-elements across *PcWOX* promoters, with light-responsive motifs most abundant and methyl jasmonate (MeJA)- and anaerobic-response elements also widespread. The promoter sequences are in Supplementary Table S5. The number and composition of elements varied between genes: *PcWOX13.3*, *PcWOX4* and *PcWOX10* carried the largest and most diverse sets of elements, while *PcWOX3.2*, *PcWUS* and *PcWOX9* had relatively few (Figure 4B). Notably, *PcWOX5* promoters were enriched for light, meristem-associated and hormone-responsive elements (MeJA, gibberellin, abscisic acid), with meristem-related motifs clustering within the −1600 to −800 bp region; by contrast, *PcWUS* promoters were dominated by light-responsive elements and included low-temperature, auxin and salicylic-acid related motifs. These promoter patterns point to differential regulation among *PcWOXs* and suggest that some members (e.g., *PcWOX5*) are primed for roles in meristem activity and hormone-mediated responses. Transient expression of *PcWOX5*-GFP and *PcWUS*-GFP in *N. benthamiana* epidermal cells produced clear nuclear fluorescence for both fusion proteins, whereas free GFP was distributed throughout the cytoplasm and nucleus. The localization results are consistent with the predicted transcriptional regulatory functions of these two *PcWOXs* (Figure 4C).

### 2.4. Identification of PcWOX-Associated Regulatory Networks by WGCNA

**Figure 5:**
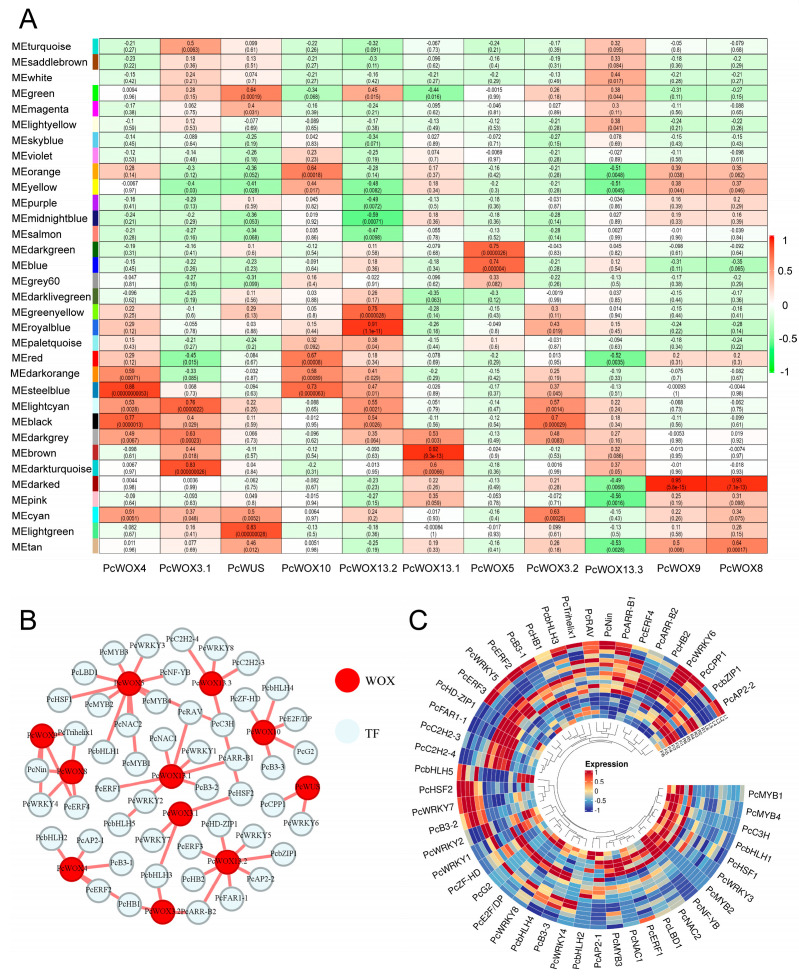
WGCNA identifies co-expression modules and putative regulatory networks associated with the *PcWOX* gene family. (**A**) Correlations between module eigengenes and the abundance profiles of the 11 *PcWOX* family members. (**B**) Weighted co-expression network showing the relationships between candidate transcription factors and hub *PcWOX* genes in representative key modules. (**C**) Transcriptional expression profiles of representative key transcription factors across different developmental stages.

To elucidate the regulatory relationships of *PcWOX* genes, WGCNA was performed using the parameters described in Section 4. Evaluation of the scale-free topology fit index and mean connectivity across candidate soft-thresholding powers indicated that β = 8 was appropriate for network construction (Supplementary Figure S1A). Using this parameter, a total of 33 co-expression modules were identified, and the overall module structure is shown in Supplementary Figures S1B and S2A. Module–trait correlation analysis, using the abundance profiles of the 11 *PcWOX* family members as traits, revealed that six modules—royalblue, steelblue, brown, darkturquoise, darkred, and lightgreen—were significantly associated with *PcWOX* abundance patterns (|r| > 0.8) and were therefore defined as key regulatory modules (Figure 5A and Figure S2B, Supplementary Table S6).

To identify candidate transcription factors associated with *PcWOX* genes, all genes from these six key modules were *integrated* and compared with the *P. cyrtonema* transcription factor database, resulting in the identification of 49 candidate TFs (Supplementary Table S7). Based on these candidates, a weighted co-expression network centered on *PcWOX* hub genes was constructed to examine their putative regulatory relationships (Figure 5B). In this network, *PcWUS* was associated with TFs such as *PcWRKY6* and *PcCPP1*, whereas *PcWOX5* showed close co-expression with members of the MYB, WRKY, LBD, bHLH, and NAC families. These results provide a useful framework for further dissecting the transcriptional regulation of growth and organogenesis in *P. cyrtonema*.

To further evaluate the potential regulatory relevance of candidate TFs, the expression patterns of representative key TFs were examined across different developmental stages (Figure 5C), and the normalized expression data for all 49 transcription factors significantly associated with *PcWOX* modules are presented in Supplementary Table S8. These TFs exhibited clear stage-dependent expression profiles, suggesting that they may be associated with the temporal regulation of *PcWOX*-associated developmental processes. A broader inspection of the normalized expression matrix further revealed pronounced tissue- and stage-dependent heterogeneity across the 18 sampled conditions (Supplementary Table S8). Among these candidates, *PcERF4*, *PcWRKY5, PcERF3*, and *PcWRKY3* showed the highest overall transcript abundance, suggesting that they may occupy relatively central positions in the *PcWOX*-associated regulatory network. Notably, *PcERF4*, *PcWRKY5*, and *PcERF3* maintained comparatively high expression across multiple stages, particularly in leaves and rhizomes, whereas *PcWRKY3* displayed a clear fibrous-root-biased pattern and reached its maximum expression at Fi4. In addition, several TFs, including *PcMYB2*, *PcC3H*, *PcMYB1*, *PcNAC2*, *PcMYB4*, and *PcARR-B2*, exhibited marked stage-dependent fluctuations, with sharp expression peaks in specific tissues or developmental stages, suggesting that they may represent candidate dynamic regulators during developmental transitions. Collectively, these patterns suggest that highly expressed TFs such as *PcERF4*, *PcWRKY5*, *PcERF3*, and *PcWRKY3*, together with strongly fluctuating TFs such as *PcMYB2, PcC3H*, *PcMYB1*, *PcNAC2*, *PcMYB4*, and *PcARR-B2*, represent priority targets for future functional studies aimed at validating their potential roles in the temporal and spatial regulation of *PcWOX*-associated growth and organogenic processes. Overall, the integrated *PcWOX* TF co-expression network provides a useful framework for further dissecting the molecular regulation of growth and organogenesis in *P. cyrtonema*.

### 2.5. Agrobacterium-Mediated Transformation and Phenotypes in N. benthamiana

**Figure 6:**
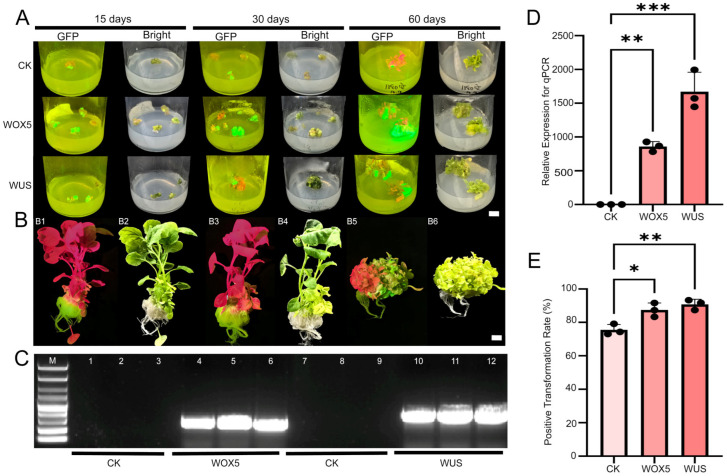
Heterologous expression of *PcWOX5* and *PcWUS* in *N. benthamiana*. (**A**) Regeneration phenotypes and GFP fluorescence of explants transformed with the empty vector, *35S::PcWOX5* and *35S::PcWUS*. (**B**) Phenotypes of regenerated tobacco plants at 85 d after transformation under white light and GFP fluorescence. (**B1**,**B2**), empty-vector control; (**B3**,**B4**), *35S::PcWOX5* regenerants; (**B5**,**B6**), *35S::PcWUS* regenerants. Red signals indicate chlorophyll autofluorescence, and green signals indicate specific GFP-tagged transgene expression. (**C**) PCR confirmation of transgene integration in regenerated plants. (**D**) Relative transcript levels of *PcWOX5* and *PcWUS* in regenerated plants, determined by RT-qPCR. (**E**) Proportion of positive regenerants in the empty-vector control, *35S::PcWOX5*, and *35S::PcWUS* groups. Positive transformants were identified by GFP fluorescence and further confirmed by PCR. Data are presented as mean ± SD. *, ** and *** indicate significant differences compared with the empty-vector control at *p* < 0.05, *p* < 0.01 and *p* < 0.001, respectively, as determined by one-way ANOVA followed by Dunnett’s multiple-comparison test after confirmation of normality and homogeneity of variance. Scale bars = 1 cm in (**A**,**B**).

To assess the regeneration-promoting activities of *PcWOX5* and *PcWUS*, their coding sequences were constitutively expressed in *N. benthamiana* using a leaf-disk transformation system. Three construct-defined groups were analyzed, namely the empty-vector control, *35S::PcWOX5* and *35S::PcWUS*. Regenerated shoots were first screened by GFP fluorescence and were subsequently confirmed by PCR. Compared with the empty vector control, both overexpression groups displayed stronger and more extensive GFP signals in regenerating tissues, with the *35S::PcWUS* explants showing the strongest fluorescence in actively proliferating shoot regions (Figure 6A).

At 85 d after transformation, white-light and fluorescence imaging revealed clear phenotypic divergence among the three groups (Figure 6B). In fluorescence images, red signals represented chlorophyll autofluorescence, whereas green signals represented specific GFP-tagged transgene expression. Empty vector regenerants showed a typical tobacco architecture, including a clear main stem, strong apical dominance, uniformly expanded broad leaves, and a well-developed fibrous root system, while no specific GFP signal was detected beyond weak background fluorescence. In contrast, *35S::PcWOX5* regenerants largely retained a recognizable main axis and normal internode elongation, but showed enhanced root proliferation. Their root systems were denser and more highly branched, and GFP fluorescence was strongly enriched in roots and weakly detectable in newly formed basal buds. By comparison, *35S::PcWUS* regenerants exhibited a much stronger reprogramming phenotype. Apical dominance was largely lost, and the plants developed into dense clusters of adventitious shoots with callus-like shoot masses. Leaves were markedly reduced in size, pale green, and tightly packed around proliferating buds, whereas root formation was strongly inhibited, with only a few short and sparse roots observed. In these plants, GFP fluorescence accumulated predominantly in newly formed meristematic regions and proliferating shoot clusters.

Molecular analyses were consistent with the phenotypic observations. RT-qPCR confirmed strong accumulation of *PcWOX5* and *PcWUS* transcripts in the corresponding regenerated lines, whereas no comparable signal was detected in the empty-vector control (Figure 6C). PCR amplification also verified stable transgene integration in positive regenerants (Figure 6D). Across three independent transformation experiments, the positive transformation rates, calculated as the mean values from three independent transformation experiments, were 75.44% for the empty-vector group, 87.43% for the *35S::PcWOX5* group, and 90.82% for the *35S::PcWUS* group (Figure 6E). In the final phenotypic dataset, 10, 30, and 30 confirmed regenerants were analyzed for the empty vector, *PcWOX5*, and *PcWUS*, which enhance regeneration competence in tobacco, but generate distinct developmental outputs, supporting their potential roles as candidate morphogenic regulators for future regeneration studies in *P. cyrtonema*.

## 3. Discussion

Previous studies have established that WOX genes are plant-specific developmental regulators that are broadly conserved across lineages, while often diverging in gene copy number, structural architecture, and recruitment into regeneration programs among crops and woody species [20,21,22]. In this study, we identified 11 *PcWOX* genes in *P. cyrtonema* and classified them into the ancient, intermediate, and modern/WUS clades, supporting conservation of the canonical WOX evolutionary framework in this medicinal monocot (Figure 2A). This overall organization is consistent with the canonical WOX family architecture reported in other plants, including woody taxa [23,24]. Comparative synteny analysis further suggested that core members such as *PcWOX5* and *PcWUS* are relatively conserved, whereas other *PcWOX* members show lineage-specific patterns consistent with differential evolutionary constraints (Figure 2B). However, the uneven chromosomal distribution, variation in exon–intron structures, and differences in promoter composition observed here also suggest that individual *PcWOX* members have undergone lineage-specific diversification after speciation (Figure 3A–E). This pattern is biologically plausible because WOX genes generally retain conserved homeodomains while diverging in regulatory sequences and expression profiles, thereby enabling functional specialization during development and regeneration [25]. Together, these results indicate that the *PcWOX* family retains a conserved structural backbone but has also undergone regulatory and architectural diversification after speciation, providing an evolutionary basis for subsequent functional specialization in *P. cyrtonema*.

Despite their conserved homeodomains, WOX paralogs often diverge markedly in spatiotemporal expression and upstream regulatory input, thereby enabling functional specialization within the family. Similar patterns have been reported in apple and other woody species, where distinct WOX homologs differ in promoter architecture, temporal expression, and regeneration-related developmental outputs, highlighting regulatory divergence as an important basis for WOX functional differentiation [23]. In our study, *PcWOX5* was preferentially expressed in fibrous roots, whereas *PcWUS* displayed a distinct expression profile more closely associated with aerial tissues and rhizome-related developmental contexts (Figure 4A), a pattern consistent with the classical distinction between WOX5-like regulators in root stem-cell niches and WUS-like regulators in shoot meristem organization. Promoter analysis further supported this divergence, as the *PcWOX5* promoter was enriched in hormone- and meristem-associated cis-elements relative to *PcWUS*, implying differences in upstream hormonal and developmental responsiveness (Figure 4B). In addition, both *PcWOX5*-GFP and *PcWUS*-GFP were predominantly localized to the nucleus (Figure 4C), supporting their identities as transcriptional regulators and suggesting that their functional divergence is more likely driven by differential regulatory deployment than by differences in subcellular localization. Collectively, by integrating expression profiling, promoter architecture, and subcellular localization, our study provides a mechanistically interpretable basis for prioritizing *PcWOX5* and *PcWUS* as regeneration-associated candidates in *P. cyrtonema*, rather than relying on phylogenetic inference alone.

Recent WOX family studies in non-model species have mainly emphasized phylogeny, conserved motifs, and basal expression patterns, whereas relatively few have combined these approaches with network-based prioritization of putative upstream regulators [10,24]. Here, WGCNA identified 33 co-expression modules, of which six were strongly associated with *PcWOX* abundance patterns (Figure 5A). A similar strategy has proven effective in other non-model medicinal plants; for example, large-scale transcriptome-based co-expression analysis in *Dendrobium catenatum* successfully reduced a broad candidate set to a small number of regulatory TFs that were subsequently validated functionally in tobacco [26]. In our dataset, the candidate TFs associated with *PcWOX* modules were mainly distributed among the WRKY, MYB, NAC, bHLH, LBD and ERF families (Figure 5B), and their normalized expression profiles further revealed substantial tissue- and stage-dependent heterogeneity. In particular, *PcERF4*, *PcWRKY5*, *PcERF3*, and *PcWRKY3* showed the highest overall transcript abundance, whereas *PcMYB2*, *PcC3H*, *PcMYB1*, *PcNAC2*, *PcMYB4*, and *PcARR-B2* displayed pronounced stage-dependent fluctuations, suggesting that *PcWOX*-associated regulation may involve both constitutively abundant and dynamically responsive TFs. These features make them particularly attractive targets for downstream functional studies (Figure 5C, Supplementary Table S8). At the same time, co-expression does not establish direct regulation, and transcriptome-scale network inference can be affected by substantial noise; therefore, these associations should be interpreted primarily as correlative and hypothesis-generating rather than mechanistically conclusive [27]. Even with this limitation, the WGCNA substantially narrows the candidate regulatory space and provides a focused framework for downstream validation. Thus, relative to a purely descriptive WOX family survey, the present study advances the field by converting transcriptome-scale breadth into a testable shortlist of candidate TFs and a *PcWOX*-centered regulatory hypothesis for future regeneration studies in *P. cyrtonema*.

WOX genes and other morphogenic regulators have been increasingly exploited to overcome regeneration bottlenecks and genotype dependence in plant transformation systems. For example, *WOX5* sustained callus proliferation and pluripotency through reciprocal regulation with SCR and activation of cell cycle-related genes, underscoring the importance of *WOX5* as a regeneration-associated regulator in plant biotechnology [28]. More broadly, regeneration engineering studies have shown that developmental regulators can substantially improve transformation performance through direct somatic embryogenesis, leaf-based transformation, and de novo meristem induction [29,30,31]. At the mechanistic level, *WOX5* and *WOX7* have been shown to maintain regenerative callus states by promoting cell division in *Arabidopsis* [32], further supporting the functional relevance of WOX activity in regeneration competence. Consistent with these studies, our heterologous assays in *N. benthamiana* showed that overexpression of *PcWOX5* and *PcWUS* enhanced regeneration-associated performance relative to the empty vector control, as supported by stronger GFP-positive regeneration signals, molecular confirmation of transgene integration and expression, and higher proportions of positive regenerants (Figure 6A,C–E). These results move the present study beyond prediction-only WOX family analysis and provide direct functional evidence that *PcWOX5* and *PcWUS* are promising candidate morphogenic regulators for future regeneration studies in *P. cyrtonema*.

An important theme in regeneration engineering is that morphogenic regulators are often beneficial only within an appropriate dosage and spatiotemporal context, whereas strong or prolonged expression can trigger pleiotropic effects, tissue damage, or regeneration failure [29,33]. This principle is well illustrated by recent work in apple, where moderate activation of *MdWUS-1* promoted leaf-borne shoot formation, but stronger activation caused rapid yellowing, browning, and explant death, suggesting that WUS activity can shift from regeneration promotion to developmental burden when expression exceeds a favorable threshold [13,34]. Similarly, transient overexpression of *ZmWus2* in *N. benthamiana* triggered broad stress, hormone, and metabolism-related reprogramming, accompanied by tissue necrosis [35]. Our results closely parallel this pattern. The phenotype of *35S::PcWUS* in tobacco was not simply enhanced regeneration; rather, it combined strong shoot-proliferative activity with severe rooting inhibition and marked developmental distortion, whereas *PcWOX5* displayed a comparatively milder and more root-associated developmental output (Figure 6B). *PcWUS* markedly enhanced shoot regeneration in the heterologous tobacco system; however, its constitutive overexpression also caused pronounced developmental alterations, including clustered shoot proliferation and reduced rooting. This pattern suggests that *PcWUS* may function as a strong but potentially pleiotropic morphogenic regulator, and that future applications may require more precise control of its expression. Although oxidative stress and hormone status were not directly measured here, the extreme clustered-shoot phenotype and impaired rooting observed in *35S::PcWUS* regenerants strongly suggest that persistent ectopic WUS activity may shift from regeneration promotion to developmental imbalance when expression is too strong or spatially uncontrolled. Accordingly, future application of *PcWUS* in *P. cyrtonema* will likely require controllable expression strategies, such as inducible or tissue-specific promoters, transgene excision systems, or transient delivery approaches, in order to balance regeneration enhancement with plant quality.

Recent reviews have emphasized that translating WOX biology into regeneration improvement requires integrating evolutionary and omics-based prioritization with functional evidence, while maintaining clear boundaries between correlative regulatory inference and direct mechanistic proof [20,27]. In this context, the present study contributes an integrated evidence chain that extends from gene family evolution and structural characterization (Figure 2 and Figure 3) to regulatory divergence inferred from expression profiling, promoter architecture, and subcellular localization (Figure 4), to network-guided prioritization of putative upstream transcription factors (Figure 5), and finally to heterologous functional evidence for regeneration-related activity (Figure 6). Taken together, these results support the conclusion that *PcWOX5* and *PcWUS* are promising candidates for regeneration-related developmental reprogramming in *P. cyrtonema* (Figure 2, Figure 3, Figure 4, Figure 5 and Figure 6). However, the current study should be interpreted within its experimental scope. Although it identifies candidate regulators and provides a testable regulatory framework for future platform development, it does not yet establish a homologous regeneration pipeline or a stable transformation system in *P. cyrtonema* itself. In addition, the WGCNA-derived regulatory relationships remain correlative rather than mechanistically validated, and the functions of *PcWOX5* and *PcWUS* were evaluated in a heterologous tobacco system under constitutive overexpression. Therefore, future work should validate both the predicted upstream regulators and the dosage-dependent morphogenic effects of *PcWOX5* and *PcWUS* in *P. cyrtonema* through direct mechanistic assays and homologous functional tests. Even with these limitations, by combining genome-wide identification, regulatory inference, and functional evidence, the present study advances *P. cyrtonema* WOX research from descriptive gene-family resources toward actionable regeneration hypotheses with practical relevance for transformation-system optimization.

## 4. Materials and Methods

### 4.1. Plant Materials and Growth Conditions

Two-year-old aseptically grown *P. cyrtonema* plantlets, collected from wild populations in Jinzhai County, Anhui Province, China, were used for gene expression assays. Plantlets were maintained at 26/22 °C (day/night) under a 16 h light/8 h dark photoperiod with a light intensity of ~50 μmol·m^−2^·s^−1^. *Agrobacterium tumefaciens* strain GV3101 (Weidi Biotechnology) served as the transformation strain. For transient assays and leaf-disk transformation, *N. benthamiana* seeds kindly provided by Yanhui Gao (State Key Laboratory for Development and Utilization of Forest Food Resources, Zhejiang A&F University, Hangzhou 311300, China) were surface-sterilized and germinated on half-strength Murashige and Skoog (½ MS) medium to obtain aseptic seedlings. No formal accession number was available for this material. Fully expanded leaves from 4 to 6-week-old seedlings were excised and used as explants. After inoculation, explants were incubated at 22–24 °C under a 16 h light/8 h dark photoperiod.

For transcriptome analysis, samples were collected from three-year-old *P. cyrtonema* plants from the same source population. Samples were collected at six developmental stages: seedling (Stage 1), leaf expansion (Stage 2), flowering (Stage 3), one month after flowering (Stage 4), early fruiting (Stage 5, 50 days post-flowering), and mid-fruiting (Stage 6, 90 days post-flowering). At each stage, rhizome (R), fibrous root (F), and leaf (L) tissues were sampled, with three biological replicates per tissue-stage combination. Samples were immediately frozen in liquid nitrogen and stored at −80 °C until further processing (Figure 1B).

### 4.2. Identification of WOX Family Members and Phylogenetic Analysis

The *P. cyrtonema* genome assembly and corresponding GFF3 annotation used in this study were generated by our group and have been deposited in the National Genomics Data Center (NGDC) under BioProject accession PRJCA021214. Reference WOX protein sequences from *A. thaliana*, *A. americana*, *A. officinalis*, and *O. sativa* were retrieved from NCBI Gene (accessed 21 May 2024). The hidden Markov model (HMM) profile of the WOX domain (PF00046) was obtained from the Pfam database (http://pfam.xfam.org/, accessed 21 May 2024), and candidate domains were verified using the NCBI Conserved Domain Database (CDD; https://www.ncbi.nlm.nih.gov/cdd/, accessed 21 May 2024).

WOX family members in *P. cyrtonema* were identified using a combination of homology-based and profile-based approaches. Briefly, HMMER v3.4 was used to search the *P. cyrtonema* predicted proteome with the WOX-domain HMM profile using an E-value cutoff of 1 × 10^−5^. Candidate sequences were further validated by BLASTP (v2.15.0) using *A. thaliana* WOX proteins as queries. Redundant sequences and truncated candidates lacking a complete WOX domain were removed after CDD verification (https://www.ncbi.nlm.nih.gov/cdd/?term= (access on 21 May 2024). The confirmed *PcWOX* protein sequences, together with WOX proteins from representative species, were then used for subsequent phylogenetic analysis. Multiple sequence alignment was performed using ClustalW in MEGA X with default parameters. Based on the aligned protein sequences, a neighbor-joining phylogenetic tree was constructed in MEGA X with 1000 bootstrap replicates.

### 4.3. Synteny Analysis of WOX Genes Among P. cyrtonema, O. sativa and A. officinalis

Genome sequences and corresponding GFF3 annotation files for *P. cyrtonema*, *O. sativa*, and *A. officinalis* were prepared. Interspecies synteny analysis was performed using the One Step MCScanX function of TBtools, and the Dual Synteny Plot module was used to generate a synteny map depicting WOX gene relationships among the three species.

### 4.4. Gene Structure, Motifs and Chromosomal Mapping

Exon-intron structures of *PcWOX* genes were extracted from the genome annotation and visualized using TBtools (v2.210). Conserved motifs in the *PcWOX* proteins were identified with the MEME suite (maximum=motifs; other parameters at default), and conserved domains were annotated using NCBI Batch CD-Search. Motifs and domains were integrated with the gene structures and visualized collectively using TBtools. Chromosomal coordinates were retrieved from the annotation and mapped on chromosomes with TBtools to produce a chromosomal distribution plot.

### 4.5. The Expression Profiles of PcWOX Genes and Promoter Analysis

Total RNA was extracted using the Mini BEST Plant RNA Extraction Kit (TaKaRa, Dalian, China) according to the manufacturer’s instructions. RNA quality and quantity were assessed using a NanoPhotometer^®^ spectrophotometer (IMPLEN, Munich, Germany), a Qubit^®^ 2.0 Fluorometer with the Qubit^®^ RNA Assay Kit (Life Technologies, Grand Island, NY, USA), and a Bioanalyzer 2100 system with the RNA Nano 6000 Assay Kit (Agilent Technologies, Santa Clara, CA, USA). In total, 54 RNA-seq libraries (6 developmental stages, 3 tissues and 3 biological replicates) were constructed using the NEB Next Ultra RNA Library Prep Kit for Illumina (New England Biolabs, Ipswich, MA, USA) and sequenced on an Illumina HiSeq 2500 platform. Raw read counts were used for differential expression analysis with DESeq, applying thresholds of |log2(fold change)| ≥ 1 and adjusted *p* value (Padj) < 0.05. For expression visualization and co-expression network analysis, read counts were normalized using the trimmed mean of M-values (TMM) method implemented in edgeR. The resulting TMM-normalized expression matrix was used to generate heatmaps in TBtools and served as the input matrix for WGCNA. For promoter analysis, 2000 bp upstream sequences from the translation start site of each *PcWOX* gene were extracted and scanned in PlantCARE to identify cis-elements. Identified elements were classified into functional categories (e.g., light-responsive, hormone-responsive, stress-related) and their distributions across promoters were visualized using TBtools. Results are provided as summary tables and distribution plots and used to infer potential regulatory divergence among *PcWOX* family members.

### 4.6. Plasmid Construction and Transient Expression

Full-length coding sequences of *PcWOX5* (531 bp) and *PcWUS* (876 bp) were amplified and fused in-frame with GFP, then recombined into the pEarleyGate101 vector under the control of the CaMV 35S promoter. Sequence-validated plasmids were introduced into *A. tumefaciens* strain GV3101 by electroporation. *Agrobacterium* cultures were grown to OD_600_ ≈ 0.5, collected by centrifugation, and resuspended in infiltration buffer (10 mM MgCl_2_, 10 mM MES, pH 5.6, and 150 μM acetosyringone). Fully expanded leaves of *N. benthamiana* were infiltrated with the suspension and incubated in darkness for 24 h, followed by 48–60 h under normal growth conditions. GFP localization was observed using a confocal laser scanning microscope.

### 4.7. Agrobacterium-Mediated Stable Transformation of N. benthamiana

Seeds of *N. benthamiana* were surface-sterilized and germinated on half-strength murashige and skoog (½ MS) medium. Fully expanded leaves from approximately 20-day-old aseptic seedlings were cut into ~0.5 × 0.5 cm leaf disks and pre-cultured on MS1 medium (MS basal salts supplemented with 30 g L^−1^ sucrose, 1.0 mg L^−1^ 6-BA, 0.1 mg L^−1^ NAA, and 7 g L^−1^ agar, pH 5.6–5.8) at 26 °C in the dark for 48 h. Leaf-disk transformation was performed following a standard co-cultivation protocol [36]. The recombinant vectors pCAMBIA1380-*PcWOX5* and pCAMBIA1380-*PcWUS*, together with the empty vector pCAMBIA1380, were introduced into *A. tumefaciens* GV3101 and verified by colony PCR. Bacterial cells were collected at OD_600_ ≈ 0.6 and resuspended in sterile MS medium to OD_600_ = 0.20–0.30. Leaf disks were immersed for 5 min, blotted dry, and co-cultured on MS1 medium in darkness for 48 h.

After co-cultivation, explants were washed with sterile solution containing 250 mg L^−1^ timentin and transferred to MS2 medium supplemented with 25 mg L^−1^ kanamycin and 250 mg L^−1^ timentin for selection and shoot induction. Cultures were maintained at 26 °C under light and subcultured every 20 d. Regenerated shoots were transferred to MS3 medium containing 100 mg L^−1^ timentin, and shoots of ~1 cm were moved to R1 rooting medium (½ MS supplemented with 0.1 mg L^−1^ NAA and 100 mg L^−1^ timentin). Approximately 2 months after co-cultivation, regenerated tissues were screened for GFP fluorescence using a handheld excitation lamp (LUYOR-3415RG; LUYOR, Shanghai, China).

For heterologous validation, three construct-defined groups were analyzed: empty vector, *35S::PcWOX5*, and *35S::PcWUS*. The transformation experiment was independently repeated three times. All confirmed positive regenerants from the three rounds were included in the final phenotypic analysis, yielding 10 plants in the empty-vector group and 30 plants each in the *35S::PcWOX5* and *35S::PcWUS* groups. Each regenerated plant was treated as one biological replicate. Positive transformants were identified by GFP fluorescence and further confirmed by PCR. The positive transformation rate was calculated as the number of confirmed positive regenerants divided by the total number of regenerants screened.

Genomic DNA was extracted using the CTAB method. Total RNA was isolated using a commercial kit, treated with DNase I, and reverse-transcribed from 1 µg RNA into first-strand cDNA. Conventional PCR was performed under standard conditions. RT-qPCR was conducted using SYBR Green chemistry with HSC70 as the internal reference gene and three technical replicates per sample. qPCR procedures followed MIQE principles, and relative expression levels were calculated using the 2^-ΔΔCt method (primer sequences in Supplementary Table S1).

Raw data were organized in Microsoft Excel 2016, and statistical analyses were performed using IBM SPSS Statistics (v26.0) and GraphPad Prism (v9.5.1). Data were first tested for normality and homogeneity of variance using the Shapiro–Wilk and Levene tests, respectively. No significant deviation from these assumptions was detected (*p* > 0.05). Phenotypic variables were analyzed by one-way ANOVA followed by Dunnett’s multiple-comparison test against the empty-vector control. Data are presented as mean ± SD, and differences were considered significant at *p* ≤ 0.05.

### 4.8. WGCNA and Module Characterization

WGCNA was performed using the WGCNA shiny toolkit (v1.0; https://github.com/shawnwx2019/wgcnashinyfun, accessed on 12 March 2024). The TMM-normalized transcriptome expression matrix described above was used for network construction. Genes with low overall expression were removed prior to analysis, and 36,294 genes were retained for subsequent network construction. To determine an appropriate soft-thresholding power, the scale-free topology fit index and mean connectivity were evaluated across candidate β values, and β = 8 was selected according to the approximate scale-free topology criterion (Supplementary Figure S1A). Based on this parameter, an unsigned adjacency matrix was constructed and transformed into a topological overlap matrix (TOM). Hierarchical clustering was then performed using the 1-TOM dissimilarity measure, and co-expression modules were identified using the blockwiseModules function with maxBlockSize = 30,000, minModuleSize = 30, and mergeCutHeight = 0.25. The resulting gene dendrogram and module assignment are shown in Supplementary Figure S1B.

The abundance data of the 11 *PcWOX* family members were used as input traits for module–trait correlation analysis. Modules showing a high absolute Pearson correlation coefficient (|r| > 0.8) with *PcWOX* abundance patterns were considered key regulatory modules. Genes from the selected modules were further compared with the *P. cyrtonema* transcription factor database to identify candidate transcription factors potentially associated with *PcWOX* genes. Weighted co-expression networks centered on *PcWOX* genes as focal hubs were visualized using Gephi (v0.10.1).

## 5. Conclusions

In conclusion, this study establishes a genome-wide framework for understanding the *WOX* gene family in *P. cyrtonema*. The *PcWOX* family combines structural conservation with clear regulatory divergence, and the WGCNA-based network further highlights candidate upstream regulators associated with *PcWOX* activity. The pronounced tissue and stage-dependent heterogeneity of these TFs, together with the identification of several highly abundant and dynamically responsive candidates, provides a focused set of targets for future dissection of *PcWOX*-associated regulatory mechanisms. Most importantly, heterologous overexpression of *PcWUS* and *PcWOX5* in *N. benthamiana* enhanced regeneration-associated performance, supporting their potential roles as candidate morphogenic regulators, with *PcWUS* showing stronger shoot-promoting activity and *PcWOX5* showing effects on root-related development. Together, these findings provide a valuable framework for future mechanistic dissection and homologous functional validation, as well as important gene resources, functional clues, and a regulatory basis for optimizing regeneration systems and genetic transformation strategies in *P. cyrtonema*. Given the increasing market demand for this species and the current limitations in efficient propagation and molecular improvement, these findings also have practical relevance for future breeding, clonal propagation, and medicinal-plant biotechnology.

## Data Availability

The raw sequencing data for *Polygonatum* species have been deposited in the National Genomics Data Center (NGDC; https://ngdc.cncb.ac.cn/?lang=en, accessed on 18 April 2026) under BioProject accession number PRJCA021214. The tissue-specific RNA-seq data covering the whole life cycle of *P. cyrtonema* are available under accession number CRA028914, and the RNA-seq data used to support genome annotation are available under accession number CRA028899. The assembled genome sequence of *P. cyrtonema* reported in this study has been deposited in the Genome Warehouse (GWH) of NGDC under accession number GWHGQKP00000000.1. All data generated or used during the study appear in the submitted article.

## Supplementary Materials

The following supporting information can be downloaded at: https://www.mdpi.com/article/10.3390/plants15111745/s1.
