# Supplementary material for: Genome-Wide Characterization of the WOX Gene Family in Polygonatum cyrtonema and Its Applications for Regeneration
Source: Plants (Basel). 2026 Jun 4;15(11):1745. doi: 10.3390/plants15111745 (PMC13259356; doi:10.3390/plants15111745)
Supplement: Supplementary file 1 [file plants-15-01745-s001.zip › Supplement figures.pptx]

## Slide 1
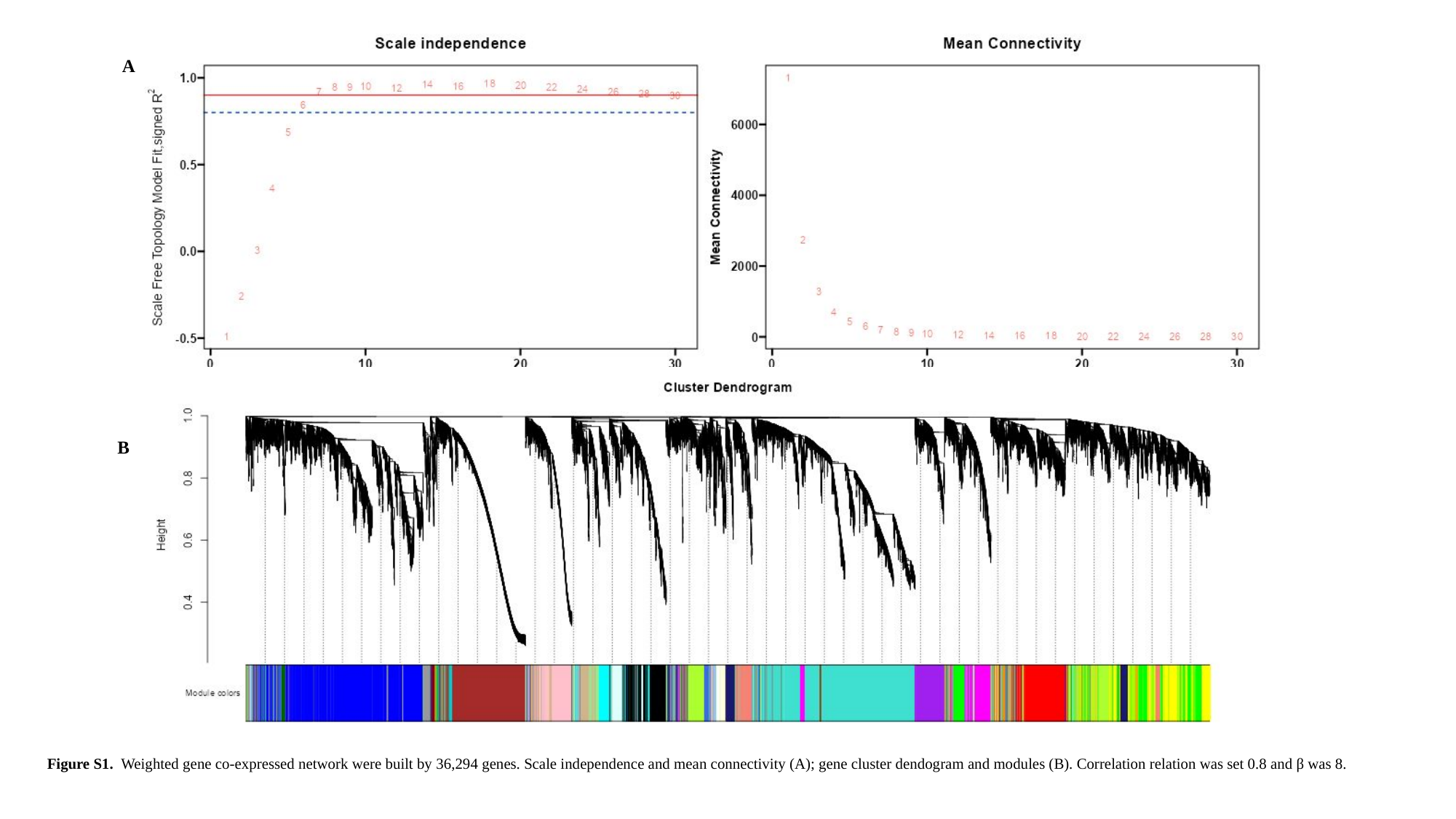

A
B
Figure S1. Weighted gene co-expressed network were built by 36,294 genes. Scale independence and mean connectivity (A); gene cluster dendogram and modules (B). Correlation relation was set 0.8 and β was 8.

## Slide 2
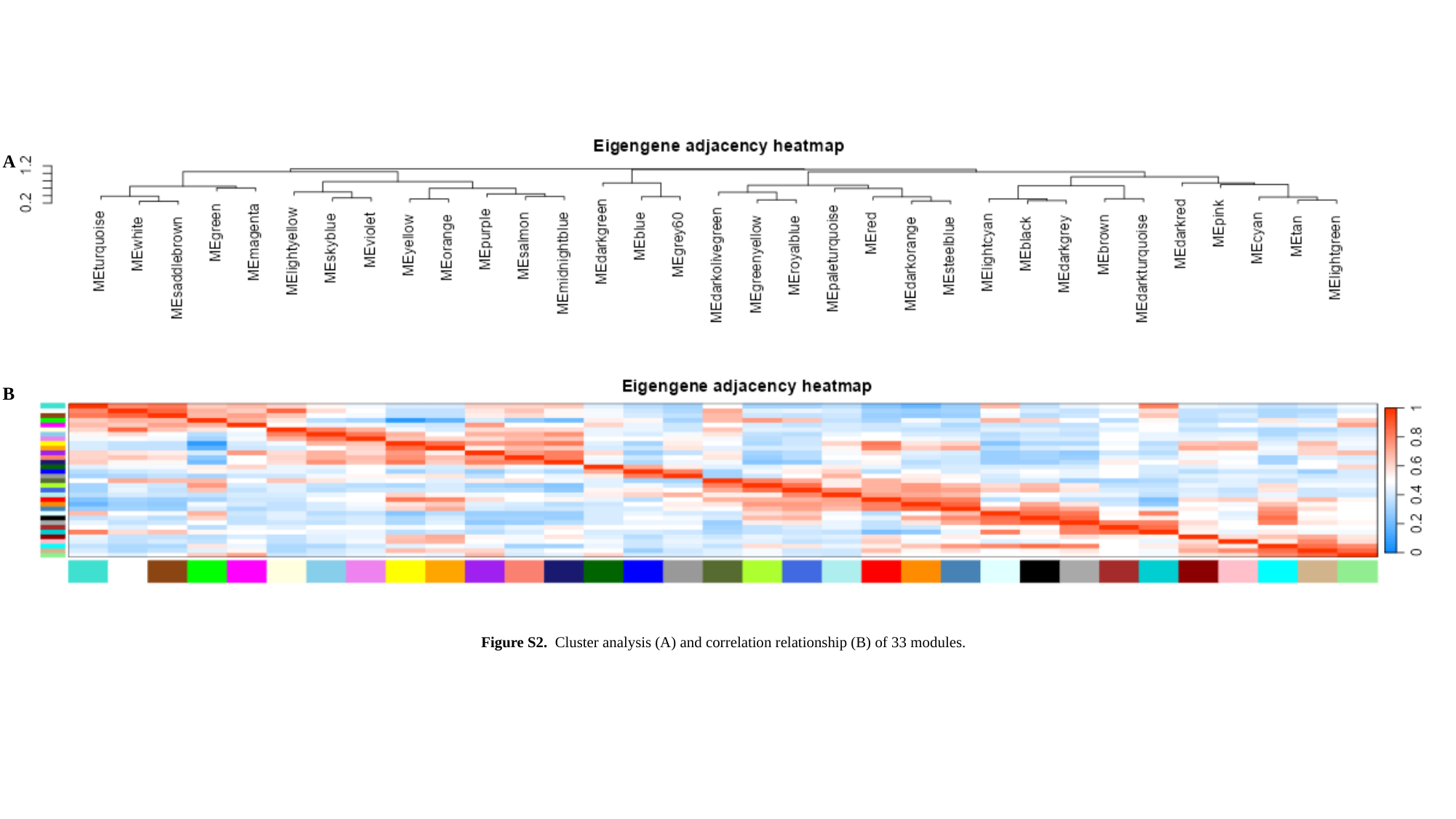

A
B
Figure S2. Cluster analysis (A) and correlation relationship (B) of 33 modules.
